# A public health value-based healthcare paradigm for HIV

**DOI:** 10.1186/s12913-021-07371-7

**Published:** 2022-01-02

**Authors:** Sebastian Vermeersch, Rémy P. Demeester, Nathalie Ausselet, Steven Callens, Paul De Munter, Eric Florence, Jean-Christophe Goffard, Sophie Henrard, Patrick Lacor, Peter Messiaen, Agnès Libois, Lucie Seyler, Françoise Uurlings, Stefaan J. Vandecasteele, Eric Van Wijngaerden, Jean-Cyr Yombi, Lieven Annemans, Stéphane De Wit

**Affiliations:** 1 Hict, Ottergemsesteenweg-Zuid 808 B/354, 9000 Gent, Belgium; 2 HIV Reference Centre, University Hospital of Charleroi, Charleroi, Belgium; 3 Department of Infectious Diseases, CHU UCL Namur, Namur, Belgium; 4 Department General Internal Medicine, Ghent University Hospital, Ghent, Belgium; 5 Department of Microbiology Immunology, Transplantation and HIV Reference Centre, University Hospital Leuven, Leuven, Belgium; 6 Department of Clinical Sciences, Institute of Tropical Medicine, Antwerp, Belgium; 7 HIV Reference Centre, Internal Medicine, C.U.B. Erasme, Université Libre de Bruxelles, Brussels, Belgium; 8 HIV Reference Centre, Universitair Ziekenhuis Brussel, Brussels, Belgium; 9 Department of Infectious Diseases and Immunity, Jessa Hospital, Hasselt, Belgium; 10 Division of Infectious Diseases, Saint Pierre University Hospital, Université Libre de Bruxelles, Brussels, Belgium; 11 HIV Reference Centre, Infectious Diseases Department, Liège University Hospital, Liege, Belgium; 12 Department of Nephrology and Infectious Diseases, AZ Sint-Jan Brugge Oostende AV, Brugge, Belgium; 13 Department of Internal Medicine and Infectious Diseases, Cliniques Universitaires Saint Luc, Brussels, Belgium; 14 Department of Public Health, Ghent University, Ghent, Belgium

**Keywords:** Value-based healthcare, HIV, Public health, Indicators, Frameworks

## Abstract

**Background:**

HIV patients face considerable acute and chronic healthcare needs and battling the HIV epidemic remains of the utmost importance. By focusing on health outcomes in relation to the cost of care, value-based healthcare (VBHC) proposes a strategy to optimize quality of care and cost-efficiency. Its implementation may provide an answer to the increasing pressure to optimize spending in healthcare while improving patient outcomes. This paper describes a pragmatic value-based healthcare framework for HIV care.

**Methods:**

A value-based HIV healthcare framework was developed during a series of roundtable discussions bringing together 16 clinical stakeholder representatives from the Belgian HIV reference centers and 2 VBHC specialists. Each round of discussions was focused on a central question translating a concept or idea to the next level of practical implementation: 1) how can VBHC principles be translated into value-based HIV care drivers; 2) how can these value-based HIV care divers be translated into value-based care objectives and activities; and 3) how can value-based HIV care objectives and activities be translated into value-based care indicators. Value drivers were linked to concrete objectives and activities using a logical framework approach. Finally, specific, measurable, and acceptable structure, process and outcomes indicators were defined to complement the framework.

**Results:**

Our framework identifies 4 core value areas where HIV care would benefit most from improvements: Prevention, improvement of the cascade of care, providing patient-centered HIV care and sustaining a state-of-the-art HIV disease management context. These 4 core value areas were translated into 12 actionable core value objectives. For each objective, example activities were proposed. Indicators are suggested for each level of the framework (outcome indicators for value areas and objectives, process indicators for suggested activities).

**Conclusions:**

This framework approach outlines how to define a patient- and public health centered value-based HIV care paradigm. It proposes how to translate core value drivers to practical objectives and activities and suggests defining indicators that can be used to track and improve the framework’s implementation in practice.

## Background

Healthcare costs are increasing due to a combination of ageing populations, a rise in prevalence of chronic diseases and a generalization of high-cost interventions. These rising costs are forcing stakeholders to consider how much and how to invest in health and healthcare. Governments look for ways to eliminate inefficient or wasteful spending and maximize value for money. At the same time, budget holders report to find it harder to achieve savings in health care than in other government spending and that they only have blunt tools available to deliver efficiency gains [1]. The result is that, in practice, increasing efficiency often translates into a simple cost-cutting exercise. This does not consider the disparate effects that simple cost cutting can have on patient outcomes across different contexts.

HIV care is one field where the pressure of managing budget versus delivering outcomes is of particular importance. Despite significant advances in combined antiretroviral therapy (cART), such as single-tablet treatment regiments, and the development of efficient prevention tools such as pre-exposure prophylaxis (PrEP), the HIV epidemic is far from over [2, 3]. Investments in HIV prevention, diagnosis, and treatment remain essential from all perspectives: the patient’s perspective, the health care budget perspective, and the societal and public health’s perspective [4]. The face of HIV care is also changing. Improved efficacy of treatment and better follow-up of patients has transformed HIV into a chronic disease with a life expectancy close to age-matched HIV-negative controls. HIV-positive individuals have a greater prevalence of multimorbidity than HIV-negative individuals and a higher number of non-infectious comorbidities, due to the duration of the HIV infection, and polypharmacy [5]. This means HIV-related health needs are increasingly important and complex [6]. To face these challenges, it is important to develop HIV healthcare paradigms that prevent new infections, ensure quality of care, deliver valuable patient outcomes, and enable efficient and effective use of limited resources.

Value-based healthcare (VBHC) offers a strategy to govern healthcare. Its aim is to optimize quality of care and cost-efficiency [7, 8]. It is based on two key principles. The first focuses on health outcomes in relation to the cost of care. The second is to consider the delivery of healthcare services across care delivery units [7]. There is no single unifying definition of what VBHC means in practice [9]. Since its inception, the concept of “value” in VBHC has shifted. Initially, Porter et al. [7, 8, 10, 11] focused on clinical outcomes and the cost-value balance associated with full cycles or episodes of care across healthcare units. Over time, the focus on efficiency, on doing “more with less resources” has been scrutinized, and more comprehensive definitions of value have been proposed. Berwick et al. focus on the patient’s individual experience of care and the cost-value balance on a population level. Sir Muir Gray proposed a paradigm shift in the analysis of value, with the aim of integrating the programming of health services to the approaches of modern population medicine [12, 13]. From a population perspective, value has three cornerstones (triple value paradigm: allocative, technical and personalized value) [14]. More recently, the Expert Panel on Effective Ways of Investing in Health (EXPH) proposed a comprehensive concept built on four value-pillars to define “value(s)-based healthcare”: allocative value, technical value, personal value, and societal value [15]. On top of these differences in conceptual approach to defining VBHC, the translation into practice can take different forms as well [9, 16–19].

The shift from efficiency to population health, and the inclusion of the societal perspective have created a perspective for the integration of prevention, equity, and a public health approach in value-based healthcare paradigms. The current paper describes a pragmatic value-based healthcare framework for HIV care. It proposes a methodological approach to build from the foundations of VBHC towards HIV-specific value drivers, considering a patient, healthcare payer, healthcare provider, and public health perspective. The pragmatic approach reflects the intent to design a framework that can realistically and sensibly be implemented in practice. It does so by a stepwise translation, each translation providing a further focus on practical considerations related to challenges specific to HIV. Out translation starts by identifying value drivers that define areas with high potential to increase the value delivered by HIV care. These drivers are further translated into objectives, activities, and, finally, quality indicators. In the discussion section, we outline how this approach addresses shortcomings in VBHC paradigms. Finally, we explain how this generalized framework can be leveraged to fuel national HIV-care plans and inform HIV health care organization.

## Methods

### Roundtable discussions leveraging a translational approach

The value-based HIV healthcare framework was developed during a series of roundtable discussions organized quarterly between June 2017 and December 2020. These roundtable discussions brought together 16 clinical stakeholder representatives from the Belgian HIV reference centers (HRC) and 2 VBHC specialists. Belgian HIV reference centers provide multidisciplinary support to HIV patients, be it medically, psychologically, familially, educationally, or socioprofessionally. HIV reference centers have access to a multidisciplinary team and the infrastructure and equipment to provide this support. The multidisciplinary team includes medical professionals (internal medicine specialists, pediatricians, gynecologists, general practitioners), non-medical therapeutic functions (nurses, social workers, clinical psychologists, dieticians, sexologists), and an administrative function. Belgian HIV reference centers are mainly financed using a system covering their non-medical therapeutic activities.

**Fig. 1:**
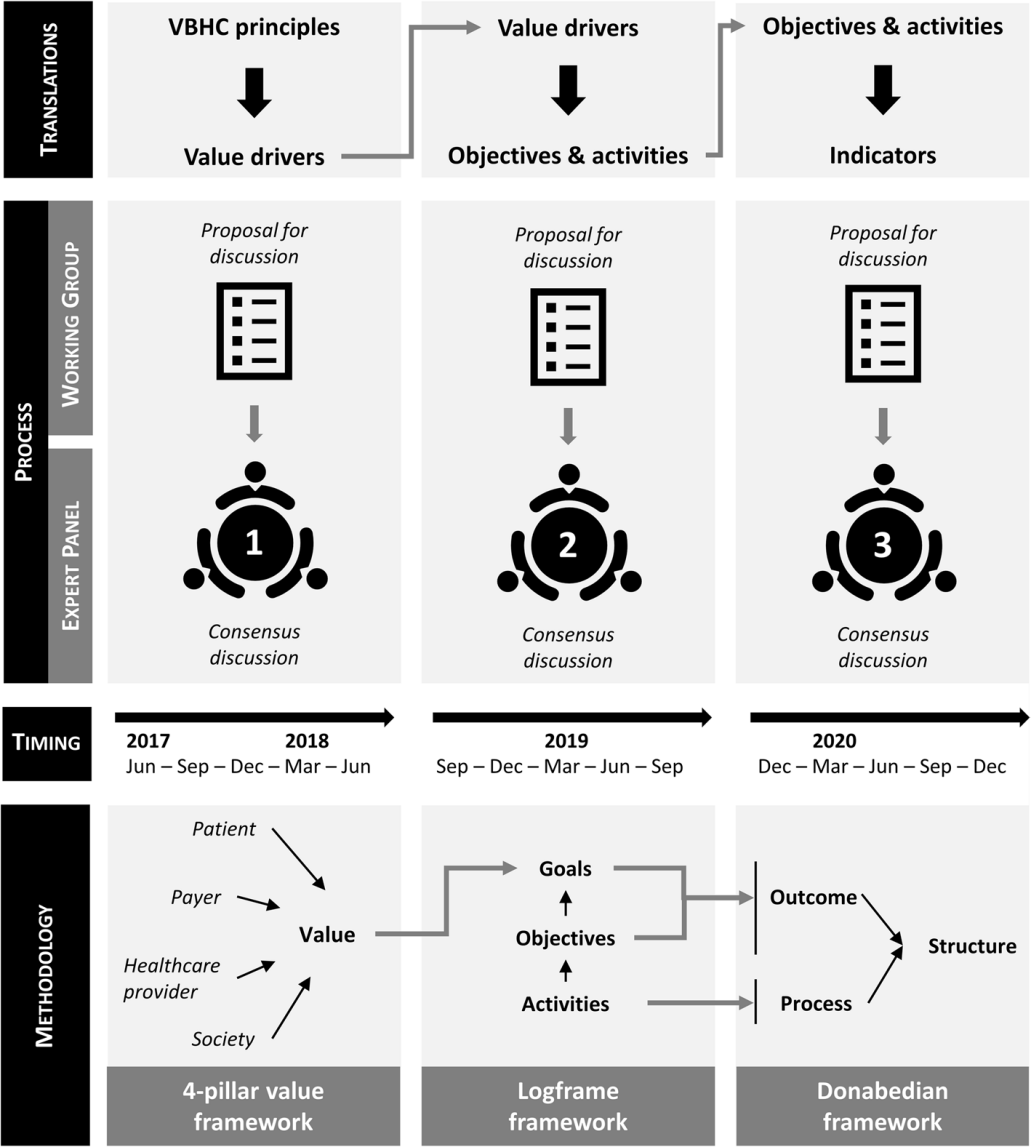
Schematic overview of the three layers of translation, the process, timelines, and methodological frameworks applied in the development of our value-based HIV healthcare framework

The roundtables were structured using a translational approach. The use of this translational approach was inspired by Bonde et al. [9], where it was used in transitioning from a DRG (Diagnosis Related Group)-financing based system to a value-based financing healthcare system in nine hospital departments in a region in Denmark. Each round of discussions was focused on a central question translating a concept or idea to the next layer of practical implementation. Three such rounds of discussion were organized (Fig. 1, top panel):

1. First translation: From VBHC principles (maximizing value for the value perspectives considered while optimizing the use of available resources) to value-based HIV care drivers.
2. Second translation: From value-based HIV care divers to value-based care objectives and activities.
3. Third translation: From value-based HIV care objectives and activities to value-based care indicators.

In preparation for each round of discussion, a working (sub)group of clinical and VBHC experts formulated a proposal for discussion. This proposal was discussed and amended over the course of a series of meetings with a broader group of experts until consensus was achieved. The consensus discussions used a nominal group technique approach [20], in which participants were first invited to prepare written feedback on the proposal for discussion, and then were invited to meet in person to discuss, and refine the discussion proposal based on these written inputs (Fig. 1, middle panel).

The methodological frameworks used for each of the three layers of translation are detailed in the sections below (Fig. 1, bottom panel).

First translation: Identifying value in HIV.

The first translation in our framework approach aims at the heart of VBHC: defining ‘value’ within the scope of HIV care and prevention. In VBHC, what constitutes value directly implies the incentives to optimize for.

The narrow focus of value in VBHC as ‘health outcomes relative to monetized inputs’ as introduced by Porter et al. [8] should be more closely looked at. From the restricted point of view of organizational units aiming to increase efficiency, it may be appropriate. From a broader public health perspective however, we argue it is not: in such an approach, there is no incentive to optimize the total ‘value’ achieved by the healthcare system [15, 21]. Our extended value-based framework is broader than the traditional but narrow interpretation of VBHC (i.e., cost-effectiveness at the healthcare provider level). It adds value at the health care provider, the healthcare system, and the public health level as well. Our framework therefore includes:

1. Value from the perspective of the *patient* (patient outcomes)
2. Value from the perspective of the *healthcare payer* and *provider* (optimized delivery of care)
3. Value from the *public health perspective* (societal value)

To be successful, our VBHC framework needs to target improving patient, healthcare payer, provider, and societal outcomes. This approach is aligned with that proposed by the EXPH (Expert Panel on Effective Ways of Investing in Health) in its opinion on “Defining value in ‘value-based healthcare’” [15].

To facilitate the definition of value, we considered key challenges in HIV care from the different perspectives, and then considered how best to tackle them. The resulting values that our framework strives to maximize were thus formulates as value drivers, each of which provide a concrete entry point to define the outcomes that need to be achieved to realize maximal impact in HIV healthcare.

### Second translation: Formulating value objectives and linked activities

The second translation focuses on linking value drivers to more specific objectives and activities. For this, we leveraged a top-down logical framework approach (logframe approach) [22]. First, each value driver was considered as a broad goal to be achieved. Next, these goals were translated into objectives that – if met – can meaningfully impact these broad goals. To achieve a goal, multiple objectives can be defined. Finally, for each objective, example activities were proposed of which the results help achieve the stated objectives. Each layer of specification (from goal to objectives and from objectives to activities) further refines the value drivers towards more concrete and specific targeted actions to take in day-to-day healthcare activities.

### Third translation: Identifying indicators

Indicators are the linchpin of a VBHC implementation. They allow to measure performance and improve implementation at each level of the framework. The final translation layer considers three types of indicators:

1. *Structure* indicators.
2. *Process* indicators.
3. *Outcome* indicators.

This approach inspired by the Donabedian-model [23, 24] identifies the requirements in the organization of care, the activities performed and the outcomes achieved to deliver VBHC. Measuring the performance of a healthcare system along these different types of indicators allows to pinpoint exactly what works or does not work in a real-world implementation. By measuring what you are attempting to improve (through the outcome indicators), by putting measurable metrics on how you are attempting to improve (process indicators), and likewise putting measurable metrics on the requirements you have identified (through the structure indicators), a comprehensive framework is provided to assess the functioning of an implementation model, and the tools have been made available to identify where to improve its implementation. As such, the approach provides a robust basis for accountability and a comprehensive starting point for continuous improvement.

Candidate indicators were scored by roundtable participants on validity, reliability, relevance, and applicability [25] and refined until participants agreed these conditions were sufficiently met.

The definition of indicators in our framework was closely related to the top-down logframe approach used in the second layer of translation. We focused first on identifying outcome indicators for each of the goals and objectives identified. Next, process indicators were linked to the activities tied to each of the objectives. We did not include structure indicators. Structure indicators cover the context in which care is delivered, including facilities, equipment, human resources as well as organizational characteristics, such as staff training and payment methods. These are the most context-specific element of our framework. Ideally, the grassroot level should define these in conjunction with the government in a retrograde way: starting from the outcome and process indicators, one can re-define the requirements for organization of care. The logframe approach can then be used in a bidirectional way to identify how to best organize and remunerate the efforts made on the field to achieve the goals described.

## Results

**Fig. 2:**
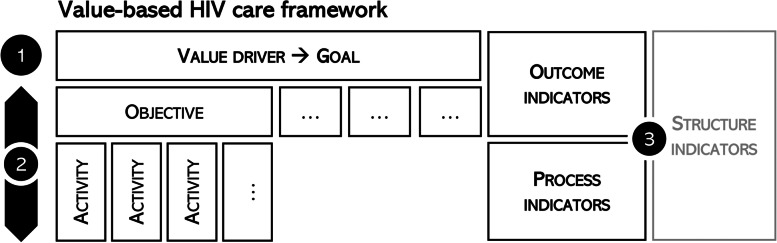
Extended value-based HIV healthcare framework. The numbers 1–3 indicate the three layers of translation performed in its development. The term extended reflects the inclusion of the public health perspective in the consideration of value (next to patient, healthcare payer, and healthcare provider value). Objectives and activities were identified using a logframe approach starting from the value drivers stated as goals to achieve. A Donabedian approach was used to identify suitable indicators at each level of the framework: outcome indicators for goals and objectives, process indicators for suggested activities. Defining structure indicators was out of scope

Figure 2 provides a schematic summary of the structure of our value-based HIV healthcare framework and the three layers of translation applied in its development.

### Key challenges and value drivers

We identified four core value areas where HIV care would benefit most from improvements:

1. *Prevention*. HIV prevention requires an integrated strategy that links prevention and care.
2. *Improvement of the cascade of care by optimizing the steps related to the care of HIV*:
  - Increase the number of people aware of their serostatus and reduce the number of undiagnosed people living with HIV (PLWH).
  - Ensure timely access to HIV care for all diagnosed PLWH.
  - Ensure prompt access to ART for all those in care and ensure achievement of an undetectable viral load for all those on ART.
  - Ensure retention in care for all those entered in HIV care.
3. *Providing patient-centered HIV health care.* The aim of patient centered HIV-care is to help provide all PLWH with the best possible quality of life. Living with an HIV infection is more than a medical issue. PLWH often also deal with a complex set of social, psychological, sexual, and other issues.
  Life expectancy for PLWH has increased. As a result, PLWH may be subject to comorbidities which are not directly related to the HIV infection. PLWH are also at increased risk of developing age-related health problems such as atherosclerosis related disease, diabetes, the metabolic syndrome, chronic kidney failure, neurocognitive diseases, and osteoporosis. It is important to prevent and manage these comorbidities.
  Maintaining sexual and reproductive health is important. Prevention, screening, and treatment of other STIs (sexually transmitted infections) is important given the interactions between HIV and other STIs. For example, the presence of an STI can increase the risk of HIV transmission and acquisition. Sexual problem and relationship issues are frequent in PLWH. Finally, prevention of mother to child transmission needs specific attention.

4. *Sustaining a state-of-the-art HIV disease management context.* Monitoring and evaluation are vital management and learning tools. Epidemiological surveillance of the HIV epidemic is essential to guide the public health priorities. Surveillance of clinical and psychosocial parameters should also be considered. Also of importance are operational and clinical research, and training programs required to educate all stakeholders concerned. A state-of-the-art disease management context relies on continuous education for healthcare professionals and providing up-to-date knowledge and training to patients so they can be empowered participants in their health and care. Finally, there is a need to implement and strengthen collaboration between all stakeholders.

Objectives, activities, and indicators

The 4 core value areas were translated into 12 actionable core value objectives that form the basis of our value-based HIV healthcare framework:

Value area 1 – Prevention:

1. Provide and support combined prevention.
2. Provide education and increase awareness
3. Provide prevention services
  Value area 2 – Improvement of the cascade of care:
4. Reduce the number of undiagnosed patients.
5. Link diagnosed patients to care.
6. Retain in care.
7. Achieve and maintain virologic control.
  Value area 3 – Providing patient-centered HIV care:
8. Support patient’s quality of life.
9. Prevent and manage comorbidities.
10. Maintain sexual and reproductive health.
  Value-area 4 – Sustaining a state-of-the-art HIV disease management context:
11. Support public health surveillance.
12. Improve knowledge through research and training.

Table 1 provides an overview of the framework with, from left to right, the 4 key value areas, the objectives per value area, and the indicators for areas, objectives and activities.

**Table 1:** Breakdown of value areas into value objectives and activities, and key indicators identified at each level of the framework. Value areas were designed to reflect patient, healthcare provider, payer, and public health perspective. Objectives and activities were identified using a logframe approach. Indicators were identified following a Donabedian model. In the framework, outcome indicators are defined at the area and objective level, process indicators are defined at the activity level

**Area**	**Objective**	*Activity*	**Indicators**	
**Prevent New Infections**				
			#	of new HIV infections
			Rate	hiv incidence per 100 000 population
	**Provide and support combined prevention**			
	PrEP			
		*Ensure that people at risk of hiv acquisition have access to PrEP*	#	of individuals who were newly enrolled on oral antiretroviral PrEP
			#	of individuals, inclusive of those newly enrolled, that received oral antiretroviral PrEP
	PEP			
		*Provide access to PEP*	#	of individuals who receive PEP
	Prevent mother-to-child transmission		%	children newly infected with HIV from mother-to-child transmission
		*Provide ART to pregnant women living with hiv*	%	Pregnant women with controlled VL
	**Provide education and increase awareness**			
	To/in target populations (MSM, migrants, PWID, …)			
		*Increase number of people informed about existing prevention measures towards HIV/STI*	%	Target population informed on existing prevention measures towards HIV and STI
	To/in the healthcare professionals			
		*Ensure that all HCPs receive training on combination prevention tools*	% and #	Of health care providers who receive training on combination prevention tools
	**Provide prevention services (condom use, counseling on risk reduction strategies, chemsex, hiv testing, …)**			
	To/in target populations (MSM, migrants, PWID, …)			
		*Increase number of people who receive prevention services*	% and #	Of target population who receive prevention services
**Improve the cascade of care**			Rate	Number of people that have died from aids-related causes per 100 000 population
	**Reduce the number of undiagnosed patients**		%	of undiagnosed PLWH
	Provide (targeted) testing		#	of tests performed annually
		*Provide access to decentralized testing*	Y/N	Availability of decentralised testing
		*Provide access to community testing*	Y/ N	Availability of community testing
		*Aim for early diagnosis*	% and #	of late diagnoses
	**Link diagnosed patients to care**		%	of diagnosed PLWH linked to care
		*Quick follow-up by reference center*	% and #	of newly diagnosed PLWH that are seen by hiv specialist within 2 weeks of diagnosis
	**Retain patients in care**		%	of PLWH retained in care
		*Re-engage patients lost-to follow-up*	% and #	of PLWH that were contacted after a standard defaulting period
			% and #	of PLWH that were re-entered in care after a standard defaulting period
		*Regular follow-up of patients*	% and #	of PLWH that have at least 1 follow-up visit in the reporting period
			#	of multidisciplinary team meetings over the course of the reporting period
	**Achieve and maintain virologic control**		%	of patients on ART with controlled viral load
		*Initiate ART treatment*	% and #	of people on ART among PLWH
		*Follow-up ART treatment*	%	of PLWH that have at least 1 measurement of VL in the reporting period
		*Follow-up ART treatment*	%	of PLWH with abnormal VL that achieve controlled VL after follow-up
**Provide Patient-Centered HIV Care**				
	**Support patient's quality of life**		%	of PLWH with good QoL as measured by standardized tool
		*Measure at least once per year QoL*	%	of patients in follow-up with QoL being measured each year
		*Provide at least once per year advice for mental wellbeing*	# and %	of patients having received support/advice for mental wellbeing
	**Prevent and manage comorbidities**		Rate	Incidence of specific comorbidities per 100 000 population
	Prevention			
		*Screening for hiv/treatment-related comorbidities*	%	of PLWH being annualy screened for hiv/treatment related comorbidities
			%	of PLWH with a smoking history documented in the last 2 years
			%	of PLWH with blood pressure recorded in the last 15 months
	Management			
		*Follow-up management of comorbidities*	# and %	of PLWH with known comorbidities
			%	of PLWH with renal function being assessed annualy
	**Maintain sexual and reproductive health**			
	Support sexual well-being & reduce risk behavior			
		*Screening for risk behaviour*	# and %	of patients in follow-up screened screened annually for risk behaviour
		*Regular asessment of sexual wellbeing*	# and %	of patients in follow-up in which sexual wellbeing is assessed annually
		*Refer patients with risky behaviour referred to prevention services*	# and %	of patients with risk behaviour referred to prevention services
		*Provide sexual wellbeing counseling*	# and %	of patients in follow-up that received sexual wellbeing counseling
	Manage and reduce hepatatis B and C infection in the hiv/aids population			
		*Provide routine hepatitis B testing*	%	of people starting ART who were tested for hepatitis B
		*Provide suitable combination therapy to hiv/hbv co-infected people*	%	of people coinfected with hiv and hbv receiving combination treatment
		*Provide routine hepatitis C testing*	%	of people starting ART who were tested for hepatitis C
		*Provide suitable combination therapy to hiv/hbc co-infected people*	%	of people coinfected with hiv and hcv having received HCV treatment in the recorded year
**Sustaining a state of the art disease management context**				
	**Support public health surveillance**			
		*Provide data for national reporting*	%	Data completion
	**Improve knowledge through research and training**			
	Provide training	Provide training		
		*Training of future HCW (for ARCs associated with teaching institutions)*	# and type	of training sessions
		*Support training of volunteers for demedicalized testing*	[Y/N]	Availability of training protocol for training non-medical staff for demedicalized testing
			#	Of accreditations awared for demedicalized training
	Participate to training			
		*Continuous (medical) education for the multidisciplinary ARC team*	#	of training sessions to which members of the multidisciplinary ARC team assisted
	Contribute / learn from scientific body of evidence			
		*Contribute to scientific publications / research*	#	of studies to which the reference center or its team members have contributed
			#	or publications (peer-reviewed / gray)
		*Active participation to scientific events/meetings*	#	of scientific meetings organized / participated to by the reference center or its team members
	Support representative organizations			
		*Organize / attend meetings with reprsensentative organizations*	#	of meetings to which reference center team members have participated

## Discussion

The concept of VBHC has gathered considerable interest since its inception by Porter and Teisberg [7]. The conceptual clarity of focusing on what matters for patients in relation to the total cost of care, provides an interesting perspective to stakeholders looking to achieve more at lower cost. Its implementation has however also triggered debate around some of its limitations. These include its narrow definition of value [12, 14, 15, 21], its hospital-centered focus on organization of care, and its neglect of important healthcare drivers such as the value of health promotion, equity, and the quality of life of citizens and healthcare professionals. Despite these limitations, the core principles of VBHC continue to be some of the most important principles driving healthcare reform in theory and practice.

Value-based healthcare paradigms have been implemented before. See [9] for the aforementioned example of an experiment in applying VBHC into healthcare governance in Denmark, [16] for a paper focusing on the development of public performance indicators with an emphasis on quality, [17] for an overview of Danish case studies, or [18] for an study exploring how representatives of four pilot teams experienced implementing VBHC in a large Swedish University Hospital over a period of two years. A notable example in HIV care is found in the Dutch OLVG hospital [26–28] that has developed and implemented a VBHC based approach to drive the organization of care for their HIV patients. In many of these examples, the focus of the VBHC implemented is on the operational aspects of care, i.e., how to optimize delivery of care in a single or small group of care center(s), without directly considering the broader public health perspective.

HIV is a prime example of a disease area suited for the application of VBHC principles. HIV care combines prevention and treatments (acute with chronic care). Even with a controlled viral load, PLWH need specific follow-up for the rest of their lives. Also, PLWH need multidisciplinary follow-up for HIV-related, HIV-treatment related, and age-related comorbidities. These characteristics make the disease area ill-suited for an activity-based healthcare financing schemes, which mostly reward volume of care over delivery of cross-functional health outcomes. In a value-based healthcare system, payment systems can be more easily structured to reimburse for value, rather than volume of care. What is particular to HIV care, and is often overlooked in VBHC paradigms, is the public health aspect. A good control of infectious diseases does not only lead to advantages for the patient (better personal health) but also to the society (less cases due to reduced onwards transmission). Any consideration of value in HIV care should include aspects that serve this public health interest.

Our value-based HIV healthcare paradigm is unique in two ways. First, in the way it combines patient, healthcare provider, payer, and public health directed value drivers. The application of this broad definition of value, which aligns closely with recent recommendations, allows to consider elements of clinical (e.g., impacting public health) and economic (e.g., considering health system financing) value explicitly. It is necessary that all stakeholders, and in particular health workers, the scientific community and institutions, undertake to promote the appropriate use of this comprehensive meaning of ‘value’ [15]. Its implementation aims to defy the inappropriate reduction of the notion of value to a purely monetary meaning. Leveraging such approach could meaningfully contribute to the long-term sustainability of the health care system. Second, our framework is unique in its approach to translate a general VBHC framework to a practical implementation level. This pragmatic approach starts from the primary focus on areas of improvement to identify the value drivers that matter most and is developed across the definition of specific objectives to help improve these outcomes, suggested activities on how these objectives can be met, and the identification of suitable outcome and process indicators to assess and direct improvement.

We see several immediate areas of application of our value-based HIV-care framework. First, it should inform national HIV plans. National HIV actions should reflect the framework’s value drivers. Second, the objectives and activities defined our framework can drive the organization of care across healthcare providers and settings. By retrograde examination of the value drivers and their associated objectives and activities, stakeholders can engage in meaningful debate on how care should be organized to meet the stated goals and objectives most effectively. Finally, the indicator set can help to transition from data collection solely for epidemiological and budgetary control, to data collection with a view to also enhance value in care.

To be successful, our framework relies on a few external factors. One crucial element already mentioned is the use of payment models that reward delivery of value in care over volume in care [29]. Various outcome-driven and mixed outcome-activity based payment models exist. The combination of a value-oriented payment system with a comprehensive definition of value can stimulate cross-healthcare collaboration. By making healthcare actors financially responsible for delivering the outcomes that matter, providing efficient pathways to deliver this value is incentivized [30]. The careful integration of outcome, process, and structure indicators can be used to direct and safeguard VBHC implementation to make sure the focus on delivering value is not lost in translation. Economic indicators could be defined to monitor and assess financial efficiency. Another external element to consider is the availability of a performant IT system to support integrated care and the measurement of costs and outcomes. This system should be easy to use, work across healthcare settings and healthcare professionals and should be well integrated in the daily clinical practice to avoid administrative overburden.

Our approach has several limitations that warrant further consideration. First, this exercise has been developed by HIV physicians and VBHC experts only, no other stakeholders have been directly involved. Notably, patient and payer representatives were not directly involved in the roundtable discussions. For the purpose of this exercise, VBHC and HIV specialists were expected to be able to represent, at least to some extent, all perspectives considered. While there are clear limitations to this approach, given the experience of participants in directly interacting with payer and patient organizations, we believe our approach still offers a valid starting point that can be further refined in future work in which these stakeholders can more explicitly be involved. Second, despite our explicit intent to generalize our concepts to be applicable outside of the direct context of its developers, our resulting framework may not be the best fit for settings with a strongly different HIV epidemiology and/or healthcare structure. The objectives and activities most suitable to deliver on the value drivers may differ if the framework approach is used by different stakeholders in different settings. Stakeholders looking to leverage our framework should validate each of our three translations in their own context and use the tools suggested to tailor the resulting value-drivers, objectives and activities and indicators. Finally, explicit economic indicators are not included in the proposed framework. Framework implementers should ideally carefully consider the economic outcome measures to include relevant to the context in which the framework is being implemented. Future work could be to develop a pilot study application of the framework that includes evaluation of its economic impact that extends to the health system and societal level.

## Conclusions

Our framework approach outlines how to define a patient- and public health centered value-based HIV care paradigm. It proposes how to translate core value drivers to practical objectives and activities and suggests defining indicators that can be used to track and improve the framework’s implementation in practice.

## Data Availability

No patient data was used and the roundtable discussions were not based on any specific dataset.

## Abbreviations

(c)ART: (Combined) antiretroviral therapy
DRG: Diagnosis related group
HCP: Healthcare professionals
HCW: Healthcare workers
HRC: HIV reference centers
Logframe: Logical framework
MSM: Men who have sex with men
PLWH: People living with HIV
PEP: Post-exposure prophylaxis
PrEP: Pre-exposure prophylaxis
PWID: People who inject drugs
STI: Sexually transmitted infection
VBHC: Value-based healthcare
